# Effects of Exercise on Inflammatory Cytokines in Patients with Type 2 Diabetes: A Meta-analysis of Randomized Controlled Trials

**DOI:** 10.1155/2020/6660557

**Published:** 2020-12-28

**Authors:** Xiaoke Chen, Xinzheng Sun, Chenghao Wang, Hui He

**Affiliations:** School of Sport Science, Beijing Sport University, 100084, China

## Abstract

**Objective:**

Inflammation is involved in the pathogenesis of type 2 diabetes (T2DM) and the occurrence of insulin resistance. The purpose of this study was to investigate the effects of exercise on inflammatory factors in patients with T2DM.

**Methods:**

A systematic review was conducted on five databases, Cochrane, Embase, Pubmed, Web of Science, and EBSCO. All randomized controlled trials (RCTs) published between establishment of the database and November 2020 without restrictions on language were included. Studies evaluated the effects of exercise intervention on inflammatory cytokines in patients with T2DM were selected.

**Results:**

Twenty-three randomized controlled trials (1350 patients) were included in our meta-analysis. Exercise can significantly reduce the level of C-reactive protein (CRP) (MD: −0.79, 95% CI: −1.26 to −0.33, *p* = 0.0008), tumor necrosis factor-*α* (TNF-*α*) (MD: −2.33, 95% CI: −3.39 to −1.27, *p* < 0.0001), and interleukin-6 (IL-6) (MD: −0.42, 95% CI: −0.60 to −0.24, *p* < 0.0001) in T2DM patients.

**Conclusion:**

The findings of this review suggest that exercise reduces inflammatory cytokines (CRP, TNF-*α*, and IL-6) in T2DM patients. More studies with high methodological qualities and large sample sizes need to be done to confirm which forms of exercise are most effective.

## 1. Introduction

Type 2 diabetes mellitus (T2DM), a chronic multifactorial disease characterized by metabolic, hormonal, epigenetic, and oxidative imbalances [1], is increasing rapidly. People with chronic diabetes are more likely to develop numerous and often serious complications including nephropathy, neuropathy, cardiovascular disease, and periodontitis [2] and may affect nearly every organ system in the body [3].

Evidence exists that inflammation is involved in the pathogenesis of T2DM and the occurrence of insulin resistance [4, 5]. Elevated circulating levels of C-reactive protein (CRP) as well as tumor necrosis factor-alpha (TNF-*α*), interleukin- (IL-) 1*β*, IL-6, and IL-1 receptor antagonist (ra) in T2DM have been described in several cross-sectional and prospective studies [6–8]. Some of the inflammatory cytokines such as IL-1*β* and TNF-*α* involved in *β*-cell damage and downregulate insulin signaling cascades in insulin-sensitive tissues, leading to the destruction of insulin sensitivity and glucose homeostasis [9–11]. Therefore, it is necessary to reduce the abnormally elevated levels of inflammatory cytokines in diabetic patients for the improvement of the disease.

Many studies have highlighted the importance of physical activity (PA) for health, and recent evidence points to positive improvements associated with exercise in T2DM [12]. Although several studies have been reported on the effects of exercise on inflammatory factors in T2DM patients, the conclusions of these studies are controversial [13, 14]. A meta-analysis on the effect of exercise on inflammatory factors in diabetic patients was published in 2013, and the results showed that exercise could reduce the levels of CRP and IL-6 [15]. However, since this paper was published in 2013, the original research data included was 10 years ago and insufficient. Costa et al. [16] also attempted to meta-analyze the impact of exercise on inflammatory factors in T2DM patients in 2016, but they did not analyze the results for the reason of lacking the original data. A large number of new researches report on the effect of exercise on inflammatory factors in T2DM nowadays, so it is necessary to make an analysis of these new studies to clarify the impact of exercise on inflammatory factors in T2DM.

The purpose of this study was to meta-analysis the data currently available on the effects of exercise on inflammatory factors in people with T2DM. Different from the study published in recent years, the innovation of our study lies in the inclusion of the latest research in the past 10 years and the addition of an analysis of TNF-*α*. Because CPR, TNF-*α*, and IL-6 are important inflammatory factors affecting the T2DM disease process [17] and the data of other inflammatory factors were insufficient, so the aim of this meta-analysis was to systematically summarize the evidence of the effects of exercise on the levels of CRP, TNF-*α*, and IL-6 in adults with type 2 diabetes.

## 2. Methods

This meta-analysis is reported in accordance with the PRISMA (Preferred Reporting Items for Systematic Reviews and Meta-Analyses) guidelines [18].

### 2.1. Data Sources and Searches

Relevant studies were identified by searching the PubMed, Cochrane, Embase, Web of Science, and EBSCO databases. For searches in PubMed/Cochrane and Embase terms from MeSH and Emtree were used, respectively. The main terms used to search for relevant publications were exercise, training, resistance training, strength training, and high-intensity interval training in combination with diabetes mellitus, diabetes, and prediabetes in combination with inflammation, cytokines, inflammat∗, and interleukin. In addition, we searched the reference lists of selected articles to identify any relevant studies that electronic searches might have missed. All randomized controlled trials (RCTs) published between establishment of the database and November 2020 without restrictions on language were included.

### 2.2. Study Selection

Studies were eligible if they (1) were RCTs, (2) included people with type 2 diabetes, (3) included a control group that did not perform exercise training, (4) compared an experimental group receiving a structured program of exercise training at least 8 weeks, and (5) assessed inflammatory factors before and after the intervention. Duplicate publications, literature review papers, letters to the editor, abstracts published in conference proceedings, studies that assessed the acute effects of a single exercise session, and animal model studies are excluded. Articles that do not have access to full text or raw data are also excluded.

### 2.3. Data Extraction and Quality Assessment

Two investigators independently abstracted all data, and the results were compiled. Disagreement was resolved by consensus or an opinion of a third author if necessary. If relevant data were unavailable in the article, we would contact the author to obtain the original data. The following information was extracted: lead author, publication year; baseline characteristics: age, sex, and number of people in the experimental group and control group; characteristics of exercise: type, frequency, duration, and intensity; and reported outcomes. We assess the risk of bias in the included studies according to the Cochrane Risk of Bias Tool [19], which includes 7 different domains as the following: (1) allocation generation, (2) concealment of allocation, (3) blinding of participants and personnel, (4) blinding of outcome assessment, (5) incomplete outcome data addressed, (6) freedom form selective reporting bias, and (7) forms of other bias.

### 2.4. Data Synthesis and Analysis

For each outcome, the prepost changes in the experimental and control groups were also pooled to estimate the effects. Mean differences (MDs) with 95% confidence intervals (CI) were calculated. We used random effects model for pooled effect estimates, which considers the variation between studies and weighs each study accordingly. Between-study heterogeneity was measured via *I*^2^ statistics and the Cochran's *Q* test. An *I*^2^ greater than 50% was interpreted as indicating substantial heterogeneity or a *p* value of 0.10 or less for the *Q* test [20]. Subanalyses were performed to determine the effect of different exercise modalities. We evaluated publication bias by inspecting funnel plots and statistically assessed the bias using the method of Egger. Sensitivity analyses were carried out to test the robustness of the pooled results by removing trials with an assessed risk of bias. The quantitative syntheses of the data were all performed with the Review Manager software, version 5.3, or the Stata software, version 12.0. For outcomes that could not be pooled, we provided a narrative summary of the findings.

## 3. Results

### 3.1. Literature Search and Trial Selection

**Figure 1:**
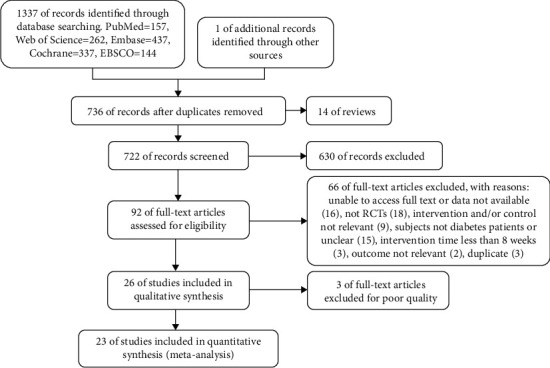
Flow chart of the study selection process.

The flow diagram reporting trial selection is shown in Figure 1. A total of 1338 potentially eligible articles were identified. After duplicates and reviews were removed, 722 articles remained for screening. By screening the titles and abstracts, 630 articles were deleted, and 92 articles were deleted after obtaining and reading the full text, leaving 26 for quantitative synthesis. Three articles were excluded due to the lack of rigorous research design, and finally, 23 studies were included in the meta-analysis.

### 3.2. Description of the Included Trials

#### 3.2.1. Participants

Included trial characteristics are summarized in Table 1. In this meta-analysis, a total of 699 patients with T2DM were included in the experimental group and 651 in the control group. There were 8 studies in which subjects had symptoms of overweight or obesity. One trial [21] included T2DM patients with coronary artery disease (CAD) and one [22] with metabolic syndrome (MS). Some studies clearly showed the male-female ratio of subjects. One study [23] separated the data of men and women, so two groups of RCT data were extracted in this study. The age of the subjects was concentrated in 40-60 years old, and most of them were middle-aged and elderly.

#### 3.2.2. Interventions

A brief description of the exercise programs is given in Table 1. One trial [22] included in the study had two experimental groups; one group was aerobic exercise, and the other group was combined exercise (aerobic exercise combined with resistance training). Therefore, two groups of RCT data were included in this study. In the study by Jorge et al. [24], a total of 3 groups of RCT data were included, in which the intervention methods were aerobic exercise, resistance training, and combined exercise. Therefore, the data included in the analysis consist of 14 groups of aerobic exercise, 5 groups of resistance training, and 7 groups of combined exercise. The major forms of aerobic exercise studied were cycling, walking, and treadmill. Most of the studies have showed the intensity of the intervention, which were concentrated in moderate to high intensity; others do not specify the intensity of intervention. The intervention time was more than 8 weeks. The control group included in the study did not take any exercise or only took regular care (not shown in the table).

#### 3.2.3. Inflammatory Outcome

All inflammatory factors studied in the included trials were counted and presented in Table 1. Most of these studies focused on CRP, TNF-*α*, and IL-6. Therefore, this meta-analysis only analyzed CRP, TNF-*α*, and IL-6. Three studies [22, 25, 26] explored the effects of exercise on IL-10 changes in diabetic patients, but two of them were from the same author. Considering that the data is relatively small, the analysis did not incorporate IL-10. Other inflammatory factors, such as IL-18, IL-15, and IL-4, have also been studied, but data about these inflammatory factors were insufficient for analysis.

### 3.3. Methodological Quality Assessment

**Figure 2:**
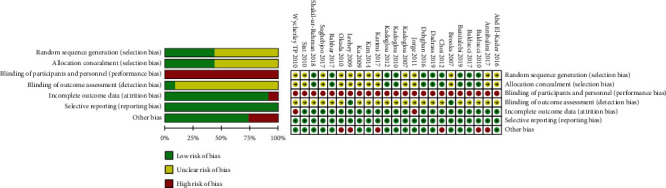
Cochrane risk bias evaluation chart.

The evaluation results of the Cochrane scale are shown in Figure 2. Ten of the included articles clearly stated the method of group allocation, and the allocation was concealed. Blinding of participants and personnel is difficult since the included experiments are all human studies; the subjects need to sign an informed consent form, and the relevant researchers need to supervise during exercise interventions. Therefore, this item is evaluated as high risk in all articles. Two articles described the use of blinding for outcome analysis. The subjects of the two articles were evaluated as high risk due to a serious loss of personnel during the intervention. Some articles clearly described the number of missing data and dealt with the missing data appropriately. No selective reporting in the included studies. Six articles were evaluated as high risk due to differences in baseline.

### 3.4. Synthesis of the Results

#### 3.4.1. Analysis of CRP

**Figure 3:**
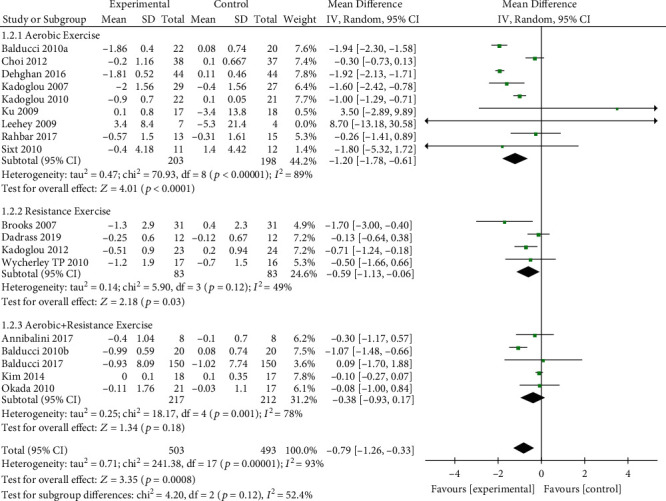
Forest plot of postintervention CRP value comparison between exercise and control groups. SD: standard deviation; Std: standardised; IV: inverse variance; CI: confidence interval.

The effect of exercise on CRP is summarized in Figure 3. We used random effects models for pooled effect estimates. 18 trials with a total of 996 participants provided data on CRP. Overall, exercise significantly declined CRP levels of -0.79 mg/l (95% CI, −1.26 to −0.33 mg/l; *p* = 0.0008; *I*^2^ = 93%; *p* for heterogeneity < 0.1). Subgroup analyses gave mixed results. The 9 studies (*n* = 403 patients) reporting CRP level for aerobic exercise programs found a significant change in CRP level of −1.20 mg/l (95% CI, −1.78 to −0.61 mg/l; *p* < 0.0001; *I*^2^ = 89%; *p* for heterogeneity < 0.1). The 4 studies (*n* = 166 patients) focusing on resistance training found a significant change in CRP level of −0.59 mg/l (95% CI, −1.13 to −0.06 mg/l; *p* = 0.03; *I*^2^ = 49%; *p* for heterogeneity = 0.12). The 5 studies (*n* = 429 patients) using a combined exercise found a nonsignificant change in CRP level of −0.38 mg/l (95% CI, −0.93 to 0.17 mg/l; *p* = 0.18; *I*^2^ = 78%; *p* for heterogeneity < 0.1).

#### 3.4.2. Analysis of TNF-*α*

**Figure 4:**
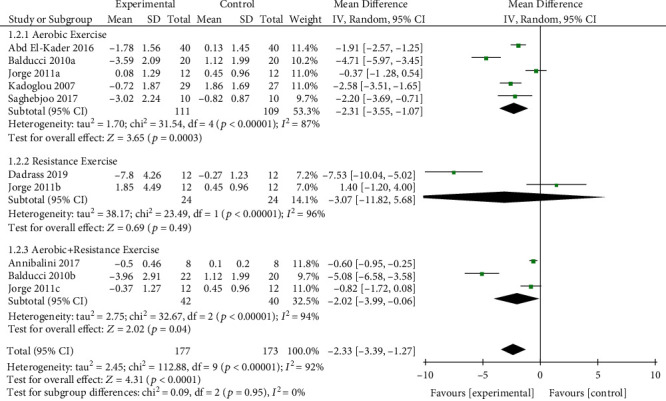
Forest plot of postintervention TNF-*α* value comparison between exercise and control groups. SD: standard deviation; Std: standardised; IV: inverse variance; CI: confidence interval.

The effect of exercise on TNF-*α* is summarized in Figure 4. We used random effects models for pooled effect estimates. Overall, exercise significantly declined TNF-*α* levels (*n* = 10 studies, 350 patients) by −2.33 *μ*g/ml (95% CI, −3.39 to −1.27 *μ*g/ml; *p* < 0.0001; *I*^2^ = 92%; *p* for heterogeneity < 0.1). In subgroup analyses, we observed a significant change in TNF-*α* levels for aerobic exercise (MD = −2.31 *μ*g/ml; *p* = 0.0003; 95% CI, −3.55 to −1.07 *μ*g/ml; *n* = 5 studies, 220 patients) and combined exercise (MD = −2.02 *μ*g/ml; *p* = 0.04; 95% CI, −3.99 to −0.06 *μ*g/ml; *n* = 3 studies, 82 patients) but nonsignificant change in TNF-*α* levels for resistance training (MD = −3.07 *μ*g/ml; *p* = 0.49; 95% CI, −11.82 to 5.68 *μ*g/ml; *n* = 2 study, 48 patients).

#### 3.4.3. Analysis of IL-6

**Figure 5:**
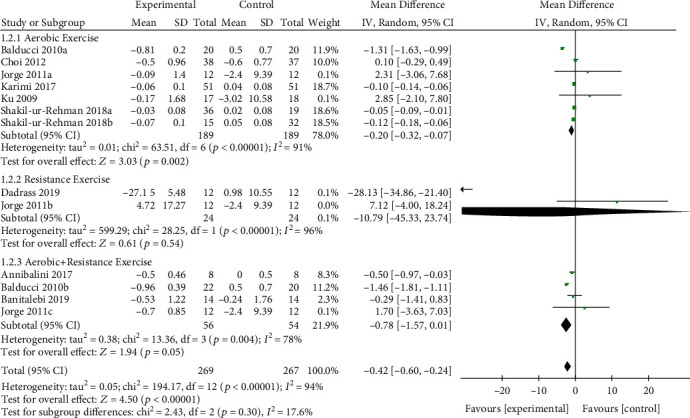
Forest plot of postintervention IL-6 value comparison between exercise and control groups. SD: standard deviation; Std: standardised; IV: inverse variance; CI: confidence interval.

The effect of exercise on IL-6 is summarized in Figure 5. We used random effects models for pooled effect estimates. 13 studies (*n* = 536 patients) examined the effect of exercise vs. nonexercise control. There was significant pooled effect estimate when assessing the efficacy of an exercise intervention for the reduction of IL-6 (MD = −0.42, *p* < 0.0001; 95% CI: −0.60 to −0.24, *I*^2^ = 94%, *p* for heterogeneity < 0.1). In subgroup analyses, the pooled effect estimates were significant for aerobic exercise (*n* = 7 studies, 378 patients) on IL-6 change (MD = −0.20, *p* = 0.002; 95% CI: −0.32 to −0.07, *I*^2^ = 91%, *p* for heterogeneity < 0.1). The pooled effect estimate was not significant for resistance exercise (*n* = 2 studies, 48 patients) on IL-6 change (MD = −10.79, *p* = 0.54; 95% CI: −45.33 to 23.74, *I*^2^ = 96%, *p* for heterogeneity < 0.1) and the combined exercise (*n* = 4 studies, 110 patients) on IL-6 change (MD = −0.78, *p* = 0.05; 95% CI: −1.57 to 0.01, *I*^2^ = 78%, *p* for heterogeneity < 0.1), respectively.

### 3.5. Sensitivity Analysis

To confirm the robustness of our results, we performed sensitivity analysis for CRP, TNF-*α*, and IL-6, respectively. After the removal of each study, sensitivity analysis of the three groups showed that the overall results were strong and stable (figure S1).

### 3.6. Evolution of Publication Bias

There was no obvious evidence of asymmetrical distribution in the funnel plot of CRP, TNF-*α*, and IL-6 levels. Egger's test was then used to further assess the publication bias. It suggests the absence of publication bias in the analysis relating CRP (*t* = −1.90, *p* = 0.076), TNF-*α* (*t* = 0.75, *p* = 0.477), and IL-6 (*t* = −0.93, *p* = 0.372) levels, respectively (figure S2).

## 4. Discussion

This systematic review summarized evidence from randomized controlled trials published in recent years and meta-analyzed the effects of exercise on the changes in inflammatory factors in T2DM patients, and it was found that exercise could significantly reduce the levels of CRP, TNF-*α*, and IL-6 in T2DM patients.

There have been numerous systematic reviews and meta-analyses showing that physical exercise can make great improvement in insulin sensitivity, increase glucose uptake in muscles and adipocytes, and reduce blood glucose levels [42, 43], but none of these recent meta-analyses systematically analyzed the long-term effects of exercise on diabetes inflammatory change. It is important to consider that the normalization of blood glucose is not sufficient to remove clinical outcomes in T2DM [44]. Studies have found that the body inflammation factor of diabetes is at a high level, making patients in a chronic low-grade inflammation state [6–8]. Therefore, reducing the body inflammation level of diabetes is of great significance for controlling and alleviating the development of diabetes. A large number of studies have been carried out on the effect of exercise on diabetic inflammatory factors [31–35], but the results are quite different. This study summarized recently published studies and found that long-term exercise can reduce the level of inflammatory factors (CRP, TNF-*α*, and IL-6) in T2DM, which may alleviate the chronic low-grade inflammation in patients to a certain extent.

CRP, TNF-*α*, and IL-6 were explored because evidences have shown a positive relationship between these inflammatory cytokines in the incidence of T2DM [24, 45, 46]. The increase of CRP may lead to apoptosis of *β*-cells by activating NF-*κ*B [47–49] and participate in insulin resistance (IR) and the pathogenesis of T2DM [50]. It could also promote endothelial cell activation and foam cell generation within the arterial wall and lead to an active participation in the pathogenesis of atherosclerosis [51]. A lot of studies showed that elevated TNF-*α* plays a direct pathogenic role in glucose metabolism and also related in *β*-cell failure [52, 53]. Further evidence indicates that TNF-*α* directly impairs peripheral insulin-stimulated glucose uptake via inhibition of Akt substrate 160 phosphorylation [54]. It was the pathological conditions associated with IR [55, 56]. Therefore, it is necessary to reduce the abnormally elevated levels of CRP and TNF-*α* in T2DM. Our study shows that exercise can significantly reduce the levels of CRP and TNF-*α* in T2DM, which can effectively reduce the inflammation state of T2DM and the damage of CRP and TNF-*α* to *β*-cells. Alleviate low-grade inflammation state can effectively prevent the aggravation of T2DM and its complications and improve the condition of T2DM to a certain extent.

Interestingly, the role of IL-6 in metabolism is debated. IL-6 is a multifunctional cytokine that plays an important role in the immune and inflammatory response, facilitating liver synthesis of acute phase proteins such as hypersensitive C reactive protein (HS-CRP) and the development of IR, and is an independent contributor to T2DM [57]. IL-6 may contribute to the pathogenesis of T2DM through interfering with the insulin signal and impairing *β*-cell function [58–60]. On the other hand, it is also released in response to physiological muscle activity as a myokine [61]. Many studies in general humans suggest that moderate acute elevations in IL-6 may inhibit TNF-*α* production and limit IL-1*β* signaling, and IL-6 related to physical activity leads to increased levels of anti-inflammatory cytokines, such as IL-1ra and IL-10 [8, 62]. It can contribute to increase peripheral insulin sensitivity [8] and improve insulin secretion [63] in healthy individuals. Some studies have suggested that exercise might reduce the IL-6 expression in the skeletal muscle and plasma levels [61, 64, 65]; these results are inconclusive according to a recent systematic review [66]. However, our study found that long-term exercise (including aerobic exercise and resistance training) resulted in a decrease in plasma IL-6 basal level in T2DM; it is essential for reducing the damage of excessive IL-6 on *β*-cell function and the deterioration of diabetic progression.

We also attempted to statistically analyze other inflammatory factors such as IL-10, IL-4, IL-8, and especially IL-1*β* (as shown in Table 1), which have been found to damage beta cells and significantly affect insulin production, but unfortunately, we found that these inflammatory factors are currently poorly studied and not sufficient in number to be included in meta-analysis.

Subgroup analysis was based on exercise type, and it was shown that aerobic exercise can significantly decrease CRP, TNF-*α*, and IL-6. And resistance training only significantly decreased TNF-*α*; combined exercise significantly declined IL-6 [24, 35]. Conflicting results were observed in the resistance training subgroup of TNF-*α* and IL-6. And there was not a significant pooled effect size favoring resistance training over control. Dadrass et al. [35] reported reduction in TNF-*α* and IL-6 serum levels in the resistance training group compared with the control group, while Jorge et al. [24] found no significant difference. However, both of them showed that resistance training favorably affects glycemic parameters. A recent systematic review concluded that both hypertrophy training and muscular endurance training exert beneficial effects on T2DM well comparable with aerobic training and that both types of resistance training can be used as potent therapeutic interventions for the management of T2DM [67]. From the perspective of inflammatory factors, it is not clear from the current results whether resistance training has a significant impact on TNF-*α* and IL-6. Thus, we recommend further studies designed with the methodological rigor necessary to prove the effectiveness of resistance training in reversing TNF-*α* and IL-6 serum levels associated with T2DM.

Jelleyman et al. [68] reported that high intensity interval training (HITT) might promote greater benefits on glucose control compared to the classical continuous aerobic exercise. However, the opposite result was reported by De Nardi et al. [69], who summarized previous studies and found no significant difference between HIIT and moderation-intensity continuous training (MICT) in improving diabetes. In fact, we also summarized the original studies of HIIT on inflammatory factors in diabetes, but the lack of original data and nonrandomized controls prevented these studies from being included in the analysis, most of which found that HIIT had no significant effects on inflammatory factors CRP, TNF-*α*, IL-6, and IL-10 [70–72].

Exercise may improve the level of inflammatory cytokines in patients with T2DM through the following mechanisms. Firstly, hyperglycemia in T2DM patients increases a variety of signaling pathways and upregulates the expression of inflammatory factors such as IL-6 and TNF-*α*. Exercise reduces the expression of inflammatory factors by improving insulin resistance and reducing blood glucose level [73]. Secondly, evidence showed that visceral fat accumulation has harmful effects on the body [74], which may exacerbate systemic inflammation and thereby activate an inflammatory pathway network, promoting the development of insulin resistance [75]. Lack of physical activity will cause the accumulation of visceral fat, thus further enhancing inflammation. Long-term physical exercise protects against accumulation of abdominal fat [76] and thereby also against chronic systemic inflammation. Thirdly, studies demonstrated that the oxidative stress induced by diabetes increased proinflammatory factors such as the level of TNF-*α* and IL-6 and raised inflammatory molecules like vascular cell adhesion molecule 1 (VCAM1) [77]. Exercise can improve oxidative stress by promoting the expression of antioxidant enzyme gene, upregulating antioxidant enzyme, increasing the bioavailability of NO, and reducing the production of ROS [78], to indirectly reduce the level of inflammatory factors.

### 4.1. Limitations

There were several limitations in the existing literature that affected the conclusions and implications of this study. First, part of the literature cannot find the original text and data, which makes it impossible to fully incorporate all existing studies into the analysis. Second, there was a low number of studies and high heterogeneity for some outcomes, which makes the interpretation and promotion of the results challenging. Third, there are few studies on the effects of different types of exercise on TNF-*α* and IL-6 in diabetes, making it impossible to draw a rigorous conclusion on the effects of different types of exercise. Finally, CRP and IL-6 have both proinflammatory and anti-inflammatory effects [79, 80], which complicates the interpretation of these results.

## 5. Conclusions and Suggestions

These systematic review and meta-analysis demonstrate that exercise reduces CRP, TNF-*α*, and IL-6 in T2DM patients. In the absence of studies on the effects of different exercise patterns on TNF-*α* and IL-6 in diabetes, rigorous conclusions cannot be drawn on the effects of different exercise patterns. Due to the fact that there are relatively few data on other inflammatory factors in the included original studies, our study only analyzed CRP, TNF-*α*, and IL-6; other inflammatory factors that affect and participate in the course of diabetes should be explored in future research. Since IL-1*β* has a similar effect to TNF-*α* in reducing insulin sensitivity [11], it is important to increase the study of the effect of exercise on IL-1*β* in diabetic patients. Other important anti-inflammatory factors such as IL-10 and IL-4 should also be further studied. There is a need to further understand the effects of various forms of exercise modalities on healthy, physically inactive adults to formulate lifestyle and physical activity recommendations to prevent chronic noncommunicable inflammatory disorders. To fully understand the anti-inflammatory effects of exercise, future research should explore the underlying molecular mechanisms that may be responsible for explaining exercise-induced reduction in inflammation.

## Figures and Tables

**Table 1:** Characteristics of the included studies.

		Control		Exercise				
Study	Patients	Sample size (M/F)	Age	Sample size (M/F)	Age	Intervening measure and intensity	Intervention duration (minutes per session, times per week, total weeks)	Inflammatory outcome
Kadoglou 2007 [25]	T2DM with obese	30 (12/18)	63.82 ± 7.03	30 (13/17)	59.33 ± 4.76	AE, intensity: 50-75% VO_2_max, treadmill, cycling, and calisthenics	45-60 min/day, 4 times/week, 6 months	TNF-a, IL-18, IL-10
Leehey 2009 [27]	T2DM with obese	6	55-81	7	55-81	AE, intensity: 45%-59% VO_2_max, treadmill	30-40 min/day, 3 times/week, 24 weeks	CRP
Ku 2009 [14]	T2DM with obese	18 F	57.3 ± 7.2	17 F	55.7 ± 6.5	AE, intensity: 4-6 metabolic equivalents, walking	60 min/day, 5 times/week, 24 weeks	IL-6, hsCRP
Choi 2012 [28]	T2DM	37 F	55.0 ± 6.0	38 F	53.8 ± 7.2	AE, intensity: 3.6-6.0 metabolic equivalents, walking	60 min/day, 5 times/week, 12 weeks	hsCRP, IL-6
Karimi 2017 [29]	T2DM	51	55.08 ± 7.67	51	53.74 ± 8.75	AE, intensity: unclear, treadmill, initiate 0 degree, increase 3 degrees per 5 weeks	10 min/day for the first 5 weeks (3 times/week), increase 30 minutes per 5 weeks, 25 weeks	IL-6
Kadoglou 2010 [26]	T2DM	21 (8/13)	60.32 ± 9.28	22 (8/14)	56.91 ± 7.09	AE, intensity: 50-80% VO_2_max, walking, jogging, and daily activities	45-60 min/day, 4 times/week, 12 months	hsCRP, IL-10, IL-18
Rahbar 2017 [30]	T2DM	15	48.60 ± 4.80	13	48.31 ± 5.02	AE, intensity: 50%-70% MHR, treadmill	30 min/day, 3 times/week, 8 weeks	CRP
Sixt 2010 [21]	T2DM with CAD	12 (8/4)		11 (10/1)		AE, intensity: 80% MHR, cycle ergometer training	90 min/day, 5 days/week, 4 weeks, follow by 30 min ergometer/day (5 days/week) plus 1 h group exercise/week, 5 months	hsCRP, adiponectin
Saghebjoo 2017 [31]	T2DM	10	49 ± 5.28	10	51 ± 3.45	AE, intensity: 65-85% MHR, jogging, running	From 15 min/day to 35 min/day, add 3-4 minutes per week, 3 times/week, 12 weeks	hsCRP, TNF-*α*
Dehghan 2016 [13]	T2DM with overweight	49	50.17 ± 5.34	49	58.83 ± 6.79	AE, intensity: 50-70% MHR, jogging	60 min/day, 3 times/week, 16 weeks	CRP
Abd El-Kader 2016 [32]	T2DM with obese	40	44.11 ± 5.89	40	43.62 ± 6.17	AE, intensity: 60-70% MHR, treadmill	25-45 min/day, 3 times/week, 12 weeks	TNF-*α*, IL-6, IL-8, leptin
Kadoglou 2012 [33]	T2DM with obese	14 (5/19)	64.6 ± 4.3	13 (7/16)	61.5 ± 5.4	RT, intensity: 60–80% 1RM	45-60 min/day, 3 times/week, 3 months	hsCRP
Brooks 2007 [34]	T2DM	31 (19/12)	66 ± 1	31 (21/10)	66 ± 2	RT, intensity: 1-8 weeks 60-80% 1RM, 10-14 weeks 70-80% 1RM	45 min/day, 3 times/week, 16 weeks	CRP, adiponectin
Dadrass 2019 [35]	T2DM	12	53.16 ± 8.12	12	54.91 ± 5.86	RT, intensity: first month 55% 1RM; second month 65% 1RM; third month 75% 1RM	50 min/day, 3 days/week, 12 weeks	CRP, TNF-*α*, IL-6
Wycherley 2010 [36]	T2DM with obese	16	55.0 ± 8.4	17	55.0 ± 8.4	RT, intensity: 70-85**%** 1RM	45 min/day, 3 days/week, 16 weeks	CRP
Annibalini 2017 [37]	T2DM	8 M	60 ± 6.8	8 M	57 ± 9.1	Combined exercise, intensity: 40-65% HRR	30-60 min/day, 3 days/week, 18 weeks	hsCRP, TNF-*α*, IL-6, MCP-1, leptin, adiponectin
Balducci 2017 [38]	T2DM	150	40-80	150	40-80	Combined exercise, intensity: unclear	60 min/day, 2 days/week, 4 months	hsCRP
Okada 2010 [39]	T2DM	17 (11/6)	46.5 ± 5.9	21 (10/11)	61.9 ± 8.6	Combined exercise, intensity: unclear	60 min/day, 3-5 days/week, 3 months	hsCRP, leptin, adiponectin
Kim 2014 [40]	T2DM with overweight or obese	17 (10/7)	48.3 ± 8.2	18 (9/9)	48.4 ± 8.6	Combined exercise, intensity: 50% 10RM for RT, 50–70% MHR for AE	70 min/day, 3 days/week, 12 weeks	hsCRP, adiponectin
Banitalebi 2019 [41]	T2DM	14	55.71 ± 6.40	14	54.14 ± 5.43	Combined exercise, intensity: 60–70% MHR for AE	3 days/week, 10 weeks	IL-6, IL-15
Shakil-ur-Rehman 2018^a/b^ [23]	T2DM	19 M	55.00 ± 8.03	36 M	59.12 ± 5.78	AE	150 min/week, 25 weeks	IL-6
		32 F	57.93 ± 6.83	15 F	51.31 ± 8.78			
Balducci 2010^a/b^ [22]	T2DM with MS	20 (9/11)	61.1 ± 7.1	20 (8/12)	64.3 ± 8.1	AE, intensity: 70-80% VO_2_max	60 min/day, 2 days/week, 12 weeks	CRP, TNF-*α*, IL-6, IL-1*β*, IL-4, IL-10
				22 (8/14)	60.6 ± 9.3	Combined exercise, intensity: 80% 1RM for RT, 70-80% VO_2_max for AE	(20RT+40AE) min/day, 2 days/week, 12 weeks	
Jorge 2011^a/b/c^ [24]	T2DM	12 (4/8)	53.42 ± 9.82	12 (5/7)	52.09 ± 8.71	AE, intensity: lactate threshold	60 min/day, 3 days/week, 12 weeks	CRP, TNF-*α*, IL-6, adiponectin
				12 (5/7)	54.10 ± 8.94	RT		
				12 (4/8)	57.90 ± 9.82	Combined exercise, intensity: half the volume of the aerobic and resistance groups		

CAD: coronary artery disease; MS: metabolic syndrome; M: male; F: female; RT: resistance training; AE: aerobic exercise; MHR: maximum heart rate; HRR: heart rate reserve; RM: repetition maximum; CRP: C-reactive protein; hsCRP: high sensitivity C-reactive protein; TNF-*α*: tumor necrosis factor-alpha; IL: interleukin; MCP-1: monocyte chemoattractant protein 1.

## Additional Points

This systematic review has been registered on PROSPERO, and the registration number is CRD42020221033.

## Supplementary Materials

Supplementary Materials Figures of sensitivity analysis and funnel plot see Supplementary Figure.
