# Supplementary material for: Effects of Exercise on Inflammatory Cytokines in Patients with Type 2 Diabetes: A Meta-analysis of Randomized Controlled Trials
Source: Oxid Med Cell Longev. 2020 Dec 28;2020:6660557. doi: 10.1155/2020/6660557 (PMC7785348; doi:10.1155/2020/6660557)
Supplement: Supplementary Materials — Figures of sensitivity analysis and funnel plot see Supplementary Figure. [file 6660557.f1.docx]

**Supplementary Figure**

**
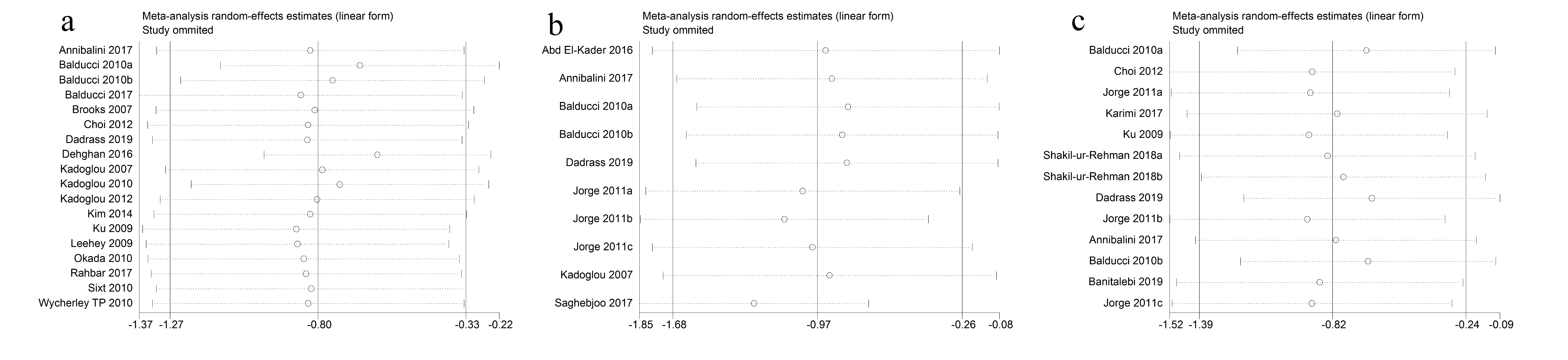
**

figure S1. Sensitivity analysis of the effects of exercise on inflammatory cytokines in patients with type 2 diabetes. (a) CRP. (b) TNF-α. (c) IL-6.

**
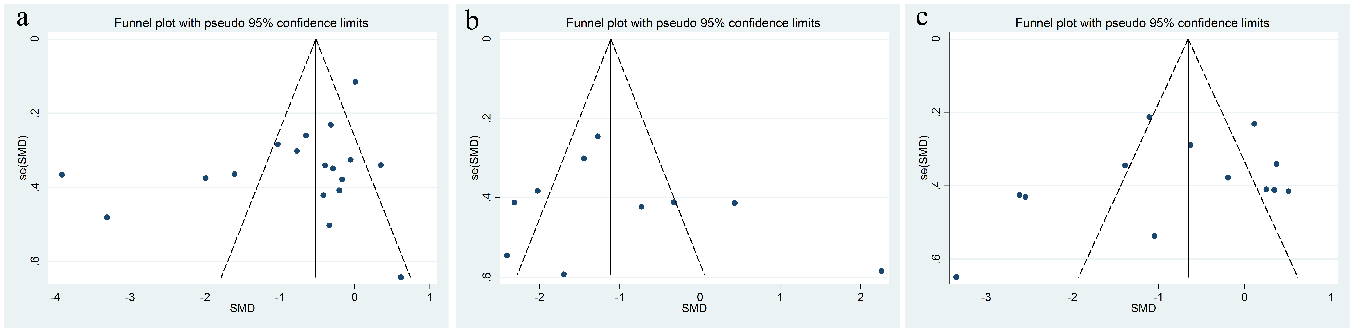
**

figure S2. Assessment of potential publicatio bias by funnel plots. (a) CRP. (b) TNF-α. (c) IL-6.
